# Pyocyanine Biosynthetic Genes in Clinical and Environmental Isolates of *Pseudomonas aeruginosa* and Detection of Pyocyanine’s Antimicrobial Effects with or without Colloidal Silver Nanoparticles

**Published:** 2012-06-13

**Authors:** Jamileh Nowroozi, Abbas Akhavan Sepahi, Afrooz Rashnonejad

**Affiliations:** 1. Department of Microbiology, Islamic Azad University, Tehran North Branch, Tehran, Iran; 2. Young Researchers Club, Islamic Azad University, Tehran North Branch, Tehran, Iran

**Keywords:** Pseudomonas aeruginosa, Pyocyanine, Phenazine Modifying Gene, Colloidal Silver Nanoparticles, Synergistic Antimicrobial Effects

## Abstract

**Objective:**

Pyocyanine plays an important role in the pathogenesis of *Pseudomonas aeruginosa, (P. aeruginosa)* and is known to have inhibitory and bactericidal effects. This study has aimed to detect the phenazine biosynthetic operon (phz ABCDEFG) and two phenazine modifying genes *(phzM and phzS)* by polymerase chain reaction (PCR) and detection of its possible protein bands by sodium dodecyl sulfate - polyacrylamide gel electrophoresis (SDS-PAGE). The antimicrobial effects of pyocyanine alone and mixed with colloidal silver nanoparticles were studied.

**Materials and Methods:**

In this descriptive study, clinical and environmental species of *P. aeruginosa* were isolated by thioglycollate medium culture and cetrimide agar, respectively. The existence of a phenazine biosynthetic operon and two phenazine modifying genes as well as their protein products were confirmed by PCR and SDS-PAGE, respectively. Pyocyanine was extracted with chloroform and its antimicrobial effects against bacteria such as; *Escherichia coli (E. coli), P. aeruginosaand Staphylococcus aureus (S. aureus)* bacteria and yeast *Candida albicans (C. albicans)* were tested using well, spot and disk diffusion methods.

**Results:**

In this study, 3 out of 48 clinical strains were unable to produce pyocyanine on cetrimide and Mueller Hinton (MH) agar. Two strains did not have phenazine modifying gene bands. Another strain did not have the possible protein band of the *phzM* gene. Pyocyanine had antimicrobial effects against the microbial strains, which increased in the presence of silver nanoparticles.

**Conclusion:**

According to the results of the present study, some *P. aeruginosa* strains are unable to produce pyocyanine due to the absence of the *phzM or phzS* genes. Therefore, these genes have an important role in pyocyanine production in *P. aeruginosa*. Pyocyanine shows synergistic antimicrobial effects in the presence of silver nanoparticles against microbial strains.

## Introduction

The gram-negative bacterium *Pseudomonas aeruginosa (P. aeruginosa)* is a ubiquitous aerobe that exists in water, soil, plants, and moist environments in hospitals. In hospitals, these bacteria are the leading cause of nosocomial lung infections and a common source of wound infections, especially inthermal burns (1). *P. aeruginosa* synthesizes a characteristic blue *redox*-active secondary metabolite, that is chloroform-soluble and a member of the tricyclic compounds "phenazine" called pyocyanine (1-hydroxy-5-methyl-phenazine) (2).One of the most important symptoms of critical infections generated by these bacteria is the production of blue pus. Pyocyanine has been shown to inhibit mammalian cell respiration (3) and the beating of human respiratory cilia *in vitro* (4). *P. aeruginosa* strains that do not produce pyocyanine have a low pathogenicity and higher susceptibility to the immune response (5). *P. aeruginosa* *PAO1* consist of two homologous core loci (opreron *phz A1B1C1D1E1F1G1* and *phz A2B2C2D2E2F2G2*) to be responsible for the synthesis of phenazine-1-carboxylic acid (PCA), and two phenazine-modifying genes (*phzS* and *phzM*) encoding enzymes involved in the conversion of PCA to pyocyanine. *phzM* encodes a protein with a calculated molecular mass of 36.4 kDa that most closely resembles bacterial O-methyl transferases. phzS encodes a protein with a molecular mass of 43.6 kDa similar to bacterial monooxygenases. Among the pseudomonads, only *P. aeruginosa*has been found to contain two copies of the phenazine operon (6).The worldwide escalation of bacterial resistance to conventional medical antibiotics is a serious concern for modern medicine (7). The antimicrobial properties of silver nanoparticles are well-established (8,9). Unique, superior, and indispensable properties of nanoparticles specifically arise from the higher surface to volume ratio and increased percentage of atoms at the grain boundaries (10). The antibacterial effects of silver salts have been reported since antiquity (11), and silver is currently used to control bacterial growth in a variety of applications, including burn wounds (12) and wound-healing (13).The cell-free culture fluid of *P. aeruginosa*, has been extensively used in the treatment of diphtheria, influenza, and meningitis during the first two decades of previous century (14). Pyocyanine also has antibiotic activity against a wide range of microorganisms (15), which may benefit P. aeruginosa by its elimination of competing microorganisms (16,17).This study aims to investigate the ability of different isolates of P.aeruginosa to produce pyocyanine as analyzed by PCR and SDS-PAGE. We also investigate pyocyanine with and without silver nanoparticles to determine if there is an increasein its antimicrobial effects (synergistic inhibition) on the growth of some bacteria and yeast.

## Materials and Methods

### Isolation and identification

In this descriptive study, wound swabs were collected from 73 patients admitted to, Shaheed Motahhari Burn Hospital, Tehran, Iran, from Dec 2009 to June 2010. Next, collected samples were cultured in thyoglycollate medium, and subcultured on nutrient agar, MacConkey agar, and eosin-methylene blue (EMB) agar plate and maintained at 37℃ for 24 hours. The isolates were identified by gram stain and biochemical tests that included oxidase, catalase, sulfide indole motility (SIM), methyl red-voges proskauer (MR-VP), triple sugar iron (TSI) agar, simon's citrate agar, nitrate broth, lysine decarboxylase, ornithine decarboxylase, arginine dihydrolase, oxidation/fermentation of glucose, growth at 42℃, as well as gelatinase and pyocyanin production according to standard microbiological methods. Soil and vegetable material samples (n=108) were collected from Karaj, Iran during the Spring of 2010. Soil samples (n=57) from14 jungles, 23 gardens, 13 agriculture farms (wheat and onion), and 7 contaminated soils with crude oil from an oil refinery were collected at a depth of 15-30 cm, combined and thoroughly mixed. Samples (n=51) from four different vegetable plants that included 11 spinach, 6 lettuces, 19 tomatoes, and 15 green onions, in addition to samples from different areas with the least hospital contamination were collected. *P. aeruginosa* was isolated by the pour plate method, using cetrimide agar as growth media. All cultures were incubated for 48 to 96 hours at 42℃ to allow for the growth of *P. aeruginosa* and inhibition of other saprophytic bacteria.

All data were collected and descriptive statistics were used for expression of data as a percentage (SPSS software Version 15.0).

### Polymerase chain reaction (PCR)

Typical colonies of P. aeroginosa were cultured in luria-bertani (LB) medium (Merck, Germany) for 24 hours at 37℃. Total DNA was isolated from 1 ml of LB culture by using a DNA purification kit (Metabion, Mi-BD 100, Germany) in accordance with the manufacturer’s instructions. Primers for pyocyanine biosynthetic genes (phz ABCDEFG operon, *phzMand phzS*) were designed in the present study (Table 1). For PCR amplification, the reaction mixture (25 µl) contained 1 µl (10 pmol/µl) of each forward and reverse oligonucleotide primersas described in table 1, 1µl of dNTP mix (200 µM; Metabion, Mi-N1006M, Germany), 2.5 µl of 10× PCR buffer (Metabion, Germany) that consisted of 15 mM MgCl_2_, 0.5 µl of Taq DNA polymerase (Metabion, Mi-E6003, Germany) and 18 µl of distilled water. Finally, 1 µl of DNA preparation (10 ng nucleic acids) was added to each 0.2 ml reaction tube. *P. aeruginosa* ATCC9027 was used as the positive control and one microtube without any DNA sample was the negative control.

All amplifications were performed by a DNA Thermal Cycler (Eppendorf Master cycler Gradient). The cycling program included a 5 minute initial denaturation step at 94℃, followed by 30 cycles of denaturation for 30 seconds at 94℃, annealing of primers for 30 seconds at 60℃, and primer extension for 30 seconds at 72℃ with auto-extension. After the last cycle, the PCR tubes were incubated for 10 minutes at 72℃. We analyzed 6 µl of the reaction mixture by standard gel electrophoresis (1% agarose); the reaction products were visualized by staining with ethidium bromide (0.5 µg/ml in the running buffer).

### Sodium dodecyl sulfate-polyacrylamide gel electrophoresis (SDS-PAGE)

A single colony of the *P. aeruginosa* strain was cultivated in 5 ml of LB medium at 37℃ for 2 days on a shaker at 200 rpm. Cells from 1/5 ml of the LB culture were harvested by centrifugation (8000 rpm for 5 minutes) and washed with 300 mM Tris, pH =8. Cell pellets were suspended in 100 µl of lysis buffer (0.32 mM sucrose, 1% SDS, 10 mM Tris-HCl, 5 mM MgCl_2_) and incubated overnight at 4℃. After incubation, unbroken cells were removed by low-speed centrifugation (8000 rpm for 5 minutes). Subsequently, sample buffer was added to the samples at a ratio of 1:4 and boiled for 4 minutes. Total proteins were separated by 12% sodium dodecyl sulfate-polyacrylamide gel (SDS-PAGE).

### Pyocyanine purification

Pyocyanine was obtained by growing *P. aeruginosa* strains in 1 liter of Mueller Hinton (MH) broth for 48 hours at 37℃ with rapid shaking. Pyocyanine was purified from bacteria medium, as previously described by Cox (18). Briefly, cells were removed by centrifugation (8000 rpm for 20 minutes). Pyocyanine was extracted from the medium with chloroform (1:0.2 ratio). Pyocyanine from the chloroform phase was extracted with 0.1 N HCl; next, we added 0.4 M NaOH to this solution until the red (acidic) pyocyanine changed to blue (basic). The pyocyanine solution was again extracted by chloroform. After five acid to base conversions the pH of the isolated acidified layer was adjusted to pH= 7.5 with a minimum volume of 0.4 M NaOH. Needlelike crystals formed in the chilled solution over the following 2 hours, trapped on a 0.45 µm (pore size) filter, washed with water, dried under vacuum, and weighed.

### Nano colloidal silver

Nano colloidal silver hydrosol (4000 ppm, water solvent) was purchased from Nano Nasb Pars Company (NanoCid). The size of the Ag-NPs was determined by transmission electron microscopy. The stock solution (1000 ppm) was used to prepare different concentrations (1-300 ppm).

### Determination of minimum inhibitory concentration (MIC), minimum bactericidal concentration (MBC), and minimum fungicidal concentration (MFC)

Antimicrobial properties were evaluated on gram-negative [*P. aeruginosa* ATCC902 and *Escherichia coli (E. coli) ATCC8739*] and gram-positive [*Staphylococcus aureus (S. aureus) ATCC6538*] bacteria and yeasts [*Candida albicans (C. albicans)* ATCC10231] by MH agar for bacteria and sabouraud dextrose agar (SDA) for yeasts.

We used inoculum prepared from a plate of 24 hour microbial culture. A suspension of microbial culture was made in sterile distilled water. The final concentration was matched to a 0.5 McFarland standard [0.5 mL of 0.048 M BaCl_2_ (1.17% m/v BaCl_2_.2H_2_O) was added to 99.5 mL of 0.18M H_2_SO_4_ (1% v/v), about 1.5×10^8^ CFU ml^-1^].

The minimum inhibitory concentration (MIC),defined as the lowest concentration of material which inhibits the growth of an organism (19), was determined by a macro dilution tube broth method, using MH broth and sabouraud dextrose broth, and a final inocula of 100 µl of 1.5 × 10^8^ CFU/ml. Different concentrations of silver nanoparticles (1-300 ppm), pyocyanine (1-300 µg ml^-1^), and a pyocyanine-silver nanoparticle compound were prepared to be tested against the microbial strains. One mL of each concentration of the antimicrobial agents were transferred to the tubes that contained the bacterial test. The contents in the tubes were mixed thoroughly and incubated overnight at 37℃. All experiments were carried out in triplicate. For growth inhibitory concentration (≥MIC), the presence of viable microorganisms was tested and the lowest concentration that caused a bactericidal effect was reported as the MBC, as suggested by Avadi et al. (20). To test for the bactericidal effect, a loopful of suspension from all tubes in which no visible bacterial growth was observed, were seeded in nutrient agar (NA) plates and then incubated overnight at 37℃. The antimicrobial concentration that caused a bactericidal effect was selected based on the absence of colonies on the agar plate.

### Determination of antimicrobial activity by the well, spot and disk diffusion tests

Antibacterial assays were performed on the microbial strains by the standard well, spot, and disk diffusion methods. Briefly, sterile MH agar and SDA were prepared for bacterial suspensions (100 µl, 1.5 × 10 8 CFU ml^-1^) and uniformly inoculated. In the disk diffusion test, sterile paper disks of 7 mm diameter (Hi-Media) were placed in each plate (5 per plate). Then, 10µl of the test compound was introduced onto the disk. For the agar well diffusion method, after the medium was solidified, 5 wells were made in the plates by a glass Pasteur pipette (7 mm). A total of 20 µl of the test compound was introduced into each well and appropriately labeled. In the agar drop diffusion test, 5 µl of each antimicrobial suspension was spotted on the medium with the bacteria. We incubated bacterial isolates at 37ºC and yeast at 25ºC for 24 hours. Zones of inhibition (diameter) produced after incubation were examined and recorded in millimeters. Varying concentrations of silver nanoparticles (1-300 ppm), pyocyanine (1-300 µg ml^-1^), and pyocyanine-silver nanoparticle compounds were used in this study.

**Table 1:** Primers designed for this study


Gene	Primer	Primer sequence(s)	PCR products

Phenazine biosynthetic operon (phzABCDEFG)	PHZP F	5´ CCGTCGAGAAGTACATGAAT 3´	448 bp
	PHZP R	5´ CATAGTTCACCCCTTCCAG 3´	
Phenazine-specific methyltransferase (phzM)	PHZP F	5´ AACTCCTCGCCGTAGAAC 3´	313 bp
	PHZP R	5´ ATAATTCGAATCTTGCTGCT 3´	
Flavine containing Monooxygennase (phzS)	PHZP F	5´ TGCGCTACATCGACCAGAG 3	664 bp
	PHZP R	5´ CGGGTACTGCAGGATCAACT 3´	


The average diameter of the inhibition zone surrounding the disk was measured by a ruler with up to 1 mm resolution. The experiment was performed in triplicate, and the mean values presented.

### Bacterial death rate determination

The kinetics of death rates of the antimicrobial agents was determined by incubation of 1.5 × 10^8^ CFU/ml of the individual bacteria in MH broth tubes. The kinetics of pyocyanine inhibition was determined according to a method by Baron et al. (15). A tube that contained 2.0 ml of MH broth was inoculated with a loop from a 48-hour trypticase soy agar plate culture of the organism to be tested. The culture was incubated for 8 to 12 hours. The contents were then used to inoculate 200 ml of MH in a 1-liter erlenmeyer flask, which was then incubated with shaking. When the cells reached the mid- to late exponential phase (absorbancy at 660 nm, 0.60 to 0.90), 18 ml samples were transferred to 125 ml erlenmeyer flasks that conatined 2 ml of pyocyanine. The cultures were incubated with shaking for 24 hours. At selected times after treatment, 0.1 ml of samples were withdrawn and diluted appropriately in 1.0% peptone. Six, 0.1 ml samples of each dilution to be plated were spread onto agar plates. The plates were incubated, and viable counts determined when growth occurred. Logarithms of the viable counts were obtained from 0 and 24 hour (s) samples of the treated cultures.

**Fig 1:**
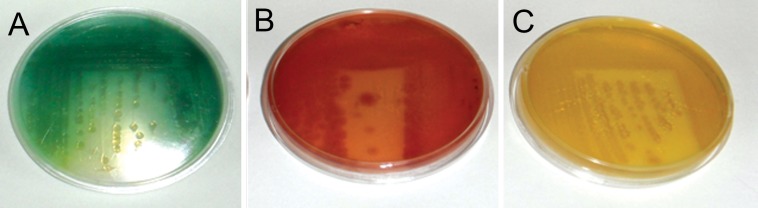
Different pigments produced by P. aeruginosa. A. P. aeruginosa E1 isolated from refinery soil. B. P. aeruginosa C6 isolated from burn wound. C. P. aeruginosa C10 isolated from burn wound.

## Results

### Clinical isolates

Table 2 shows the number and percent of *P. aeruginosa* isolated from burn wounds, soil and plant samples. Three (6.25%) strains of clinical isolates did not produce the blue-green color on cetrimide, MH, and nutrient agars. On these culture media, 2 (4.17%) isolates showed a yellow color and 1 (2.08%) isolate was a reddish-brown color (Fig 1).

### Environmental isolates

**Fig 2:**
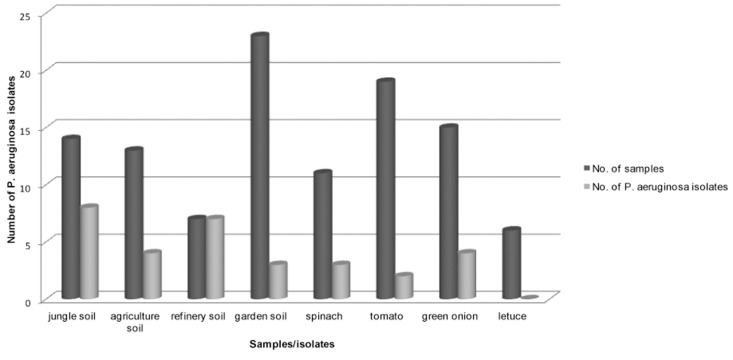
Correlation between numbers of environmental samples and P. aeruginosa isolates.

Table 2 and figure 2 show the numbers of *P. aeruginosa* isolated from different environmental sources. Total strains of non-clinical isolates produced a blue-green color on cetrimide agar. Refinery soil had more *P. aeruginosa* bacterium than the other soils; these strains produced the maximum pigment in less time, such that all the cetrimide plates became a blue-green color during 24 hours.

**Table 2:** Percentage of P. aeruginosa according to source of isolation


Source of isolation	Specimens	No. of P. aeruginosa isolates	Isolates (%)

Burn	73	48	65.75
Soil	57	23	40.35
Plant	51	9	17.06
Total	181	80	44.19


### Detection of phenazine biosynthetic operons, phzM, and phzS genes in P. aeruginosa strains

**Fig 3:**
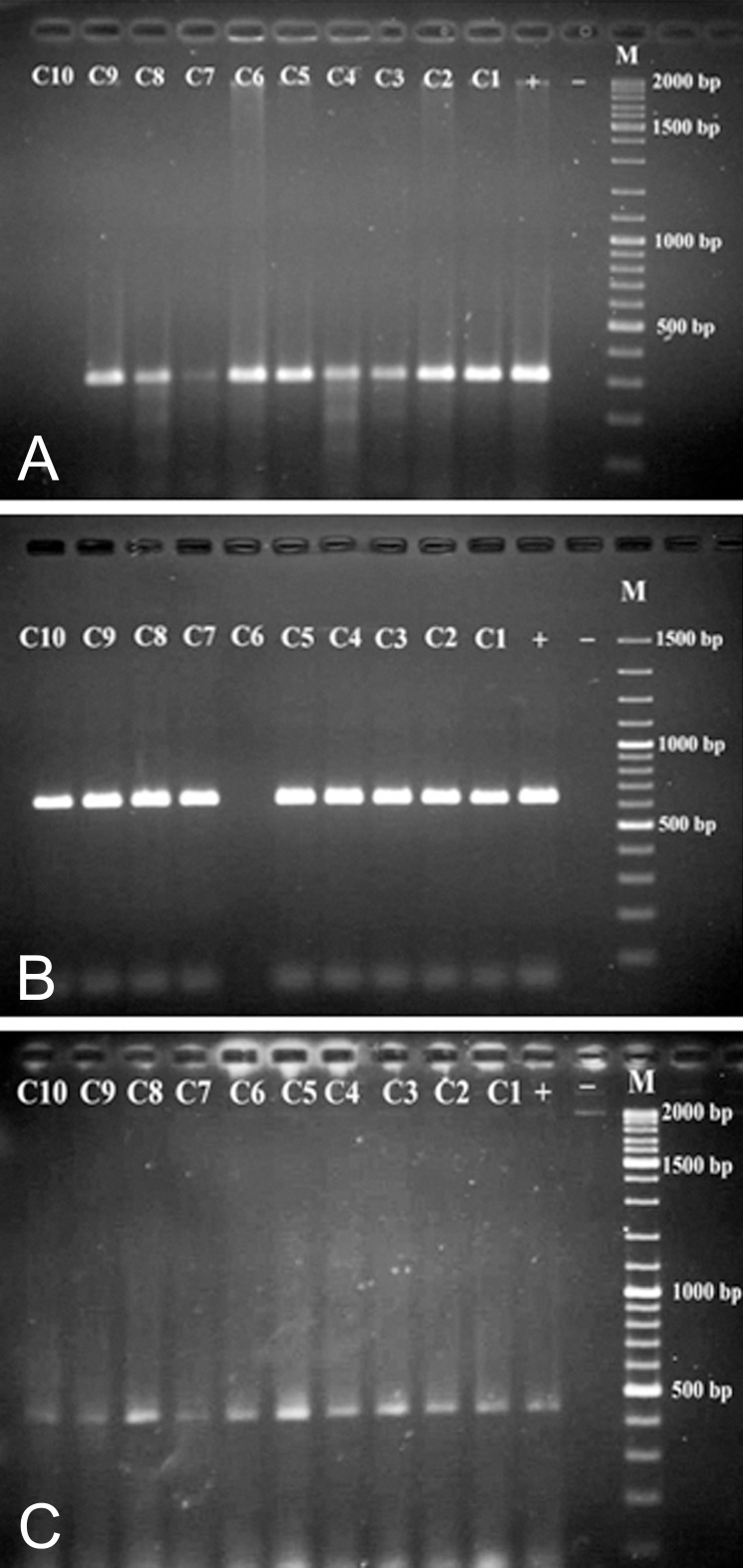
Agarose gel electrophoresis of the PCR products amplified from genomic DNA of isolated P. aeruginosa strains with primers PHZM-F and PHZM-R (A), PHZS-F and PHZS-R (B), and PHZP-F and PHZP-R (C). Lane M, DNA ladder marker. Lane -, negative control; Lane +, positive control (P. aeruginosa ATTC9027), lanes C1 to C10: P. aeruginosa isolated from burn wounds.

A PCR assay was developed to detect the phenazine biosynthetic operons, and *phzM* and *phzS* genes in *P. aeruginosa* strains by PHZP-F and PHZP-R, PHZM-F and PHZM-R, and PHZS-F and PHZS-R primers, respectively. When amplified fragments were separated on agarose gel electrophoresis (1%), only the 448 bp band of the phenazine biosynthetic operon was found in all strains, which was isolated from the clinical and environmental samples. The 313 bp and 644 bp band, which corresponded to the *phzM* and *phzS* genes, was not found in clinical strains C10 and C6, respectively (Fig 3). C10 strain produced a yellow color and C6 strain produced a reddish-brown color. To confirm the PCR results, cellular proteins of *P. aeruginosa* were separated by SDS-PAGE.

### Detection of protein bands encoded by phzM and phzS

**Fig 4:**
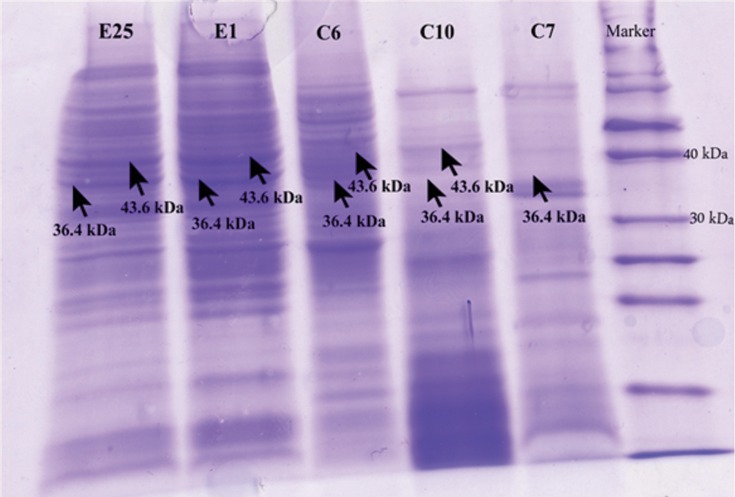
SDS–PAGE of expressed proteins. Total proteins were isolated from 3 clinical (C6, C7, C10) and 2 environmental strains (E1, E25) of *P. aeruginosa*. Lanes C7 and C10 lacked 36.4 kDa and lane C6 lacked the 43.6 kDa band related to phzM and phzS, respectively. Lanes E1 and E25 showed these protein bands.

Cellular protein of *P. aeruginosa* was detected on 12% SDS-polyacrylamide gel. C10 and C6 strains that did not have 313 bp and 644 bp DNA fragments lacked the protein bands of about 36.4 kDa and 43.6 kDa, respectively. In another clinical strain (C7) that produced a yellow color on cetrimide agar, with SDS-PAGE, a 36.4 kDa protein band was not detected. However this strain had a 313 bp band that was related to the *phzM* gene in PCR (Fig 4). All environmental strains had these protein bands on SDS-gel.

### Anti-microbial activity pyocyanine

We obtained 4 mg pyocyanine from 1 L of MH broth, inoculated with *P. aeruginosa*. Table 3 shows the MIC, MBC, and MFC values of pyocyanine, colloidal silver nanoparticles and pyocyanine-silver nanoparticle mixture against the microbial strains. The results of the spot diffusion, well and disk diffusion methods were compared with broth macrodilution MICs (Table 4). Growth of *S. aureus* and E. coli were inhibited at almost the same concentration of pyocyanine (MIC=56) but the spot, well and disk diffusion inhibition zones of S. aureus were slightly larger than *E. coli. C. albicans* was more resistant than the bacteria to pyocyanine. *P. aeruginosa*, the producer of pyocyanine, was totally resistant to the level of tested pyocyanine.

**Table 3:** MIC and MBC or MFC (µg/mL) of colloidal silver nanoparticles, pyocyanine and mixture of pyocyanine-colloidal silver nanoparticles for various microorganisms


Bacterial isolates	Colloidal silver nanoparticles	Pyocyanin	Mixture

S. aureus	3.5(3.5)	56(56)	2-25(2-25)
E. coli	6(8)	56(56)	4-25(4-25)
P. aeruginosa	9.5(13)	R(R)	*
C. albicans	12(20)	100(120)	4-50(4-50)


*No synergistic antimicrobial effects on P. aeruginosa. R: Resistant.

**Table 4:** Zones of inhibition (mm) of different antibiotics against test strains (in presence of silver nanoparticles, pyocyanine and mixture of pyocyanine-colloidal silver nanoparticles at 100 ppm, 100 µg/mL, and mixture of both, respectively


Bacterial isolates	Colloidal silver nanoparticles	Pyocyanin	Mixture

S. aureus	19/22.5/27	14/15.5/21	26/28/38.5
E. coli	15.5/17/23.5	12/14/19	21.5/23/29.5
P. aeruginosa	13.5/15/19.5	R/R/R	11/13/14.5
C. albicans	12.5/13.5/16	10.5/12/14.5	15.5/16.5/19.5


R: Resistant.

### Colloidal silver nanoparticles

**Fig 5:**
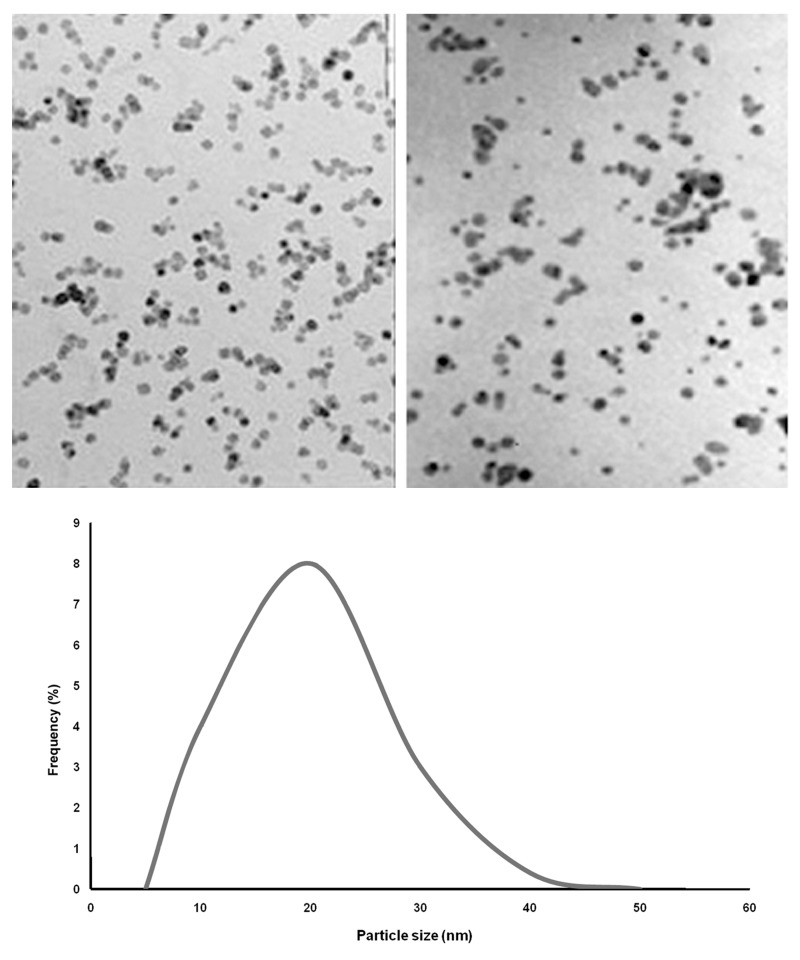
Transmission electron micrographs of colloidal silver nanoparticles. Particle size histogram of the silver particles is show his picture (Note: All scale bars are equivalent to 1 µm).

Transmission electron microscopy images recorded from the silver nanoparticles are shown in figure 5. Histograms of the silver particles (below illustration in Fig 5) showed that the particles ranged in size from 5 to 50 nm (average size: 20 nm). The MIC, MBC, and MFC values of all microbes towards colloidal silver nanoparticles are shown in Table 3. The MIC and MBC values ranged from 3.5 to 12 ppm (average: 3.5 ppm for MIC and 20 ppm for MBC). The results showed good inhibitory and bactericidal effects of the colloidal silver nanoparticles against the tested microbes. *S. aureus* was the most susceptible (MIC: 3.5 ppm) and *C. albicans* was the most resistant (MIC: 12 ppm) to this antimicrobial agent. Results of MIC, MBC, and MFC were confirmed by the spot, well and disk diffusion tests of colloidal silver nanoparticles. *P. aeruginosa* resistant strains were observed at dilutions of 20 ppm to 100 ppm.

### Pyocyanine mixed with colloidal silver nanoparticles

**Fig 6:**
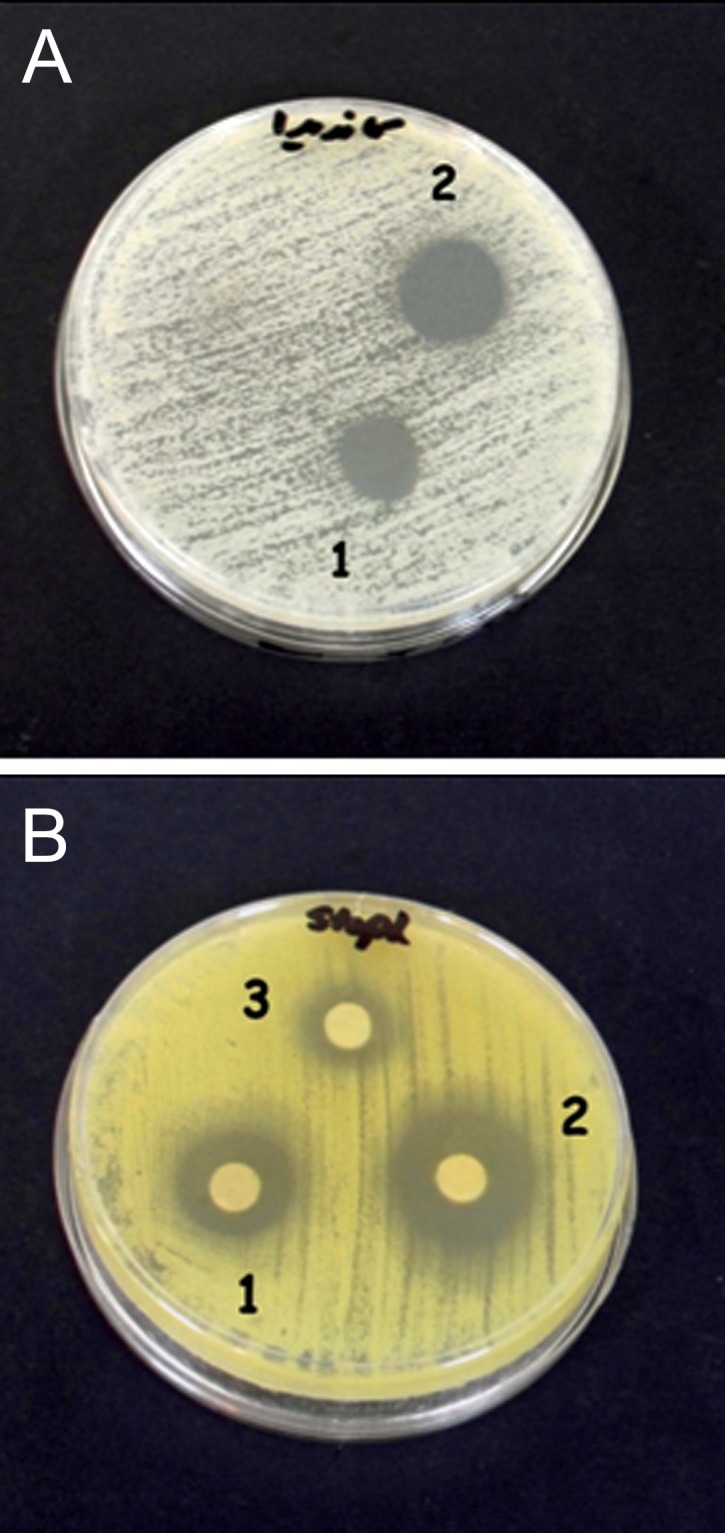
Antimicrobial effects of 100 ppm colloidal silver nanoparticles (1), mixture of pyocyanine and colloidal silver nanoparticles (2) and 100 µg/ml pyocyanin (3) on C. albicans (A) and S. aureus (B).

Pyocyanine associated with colloidal silver nanoparticles exhibited an additive effect. The combination of colloidal silver and pyocyanine showed a high increase in antibiotic synergy against gram-positive bacteria. The MIC of pyocyanine was the same for *S. aureus* and *E. coli*, however with the addition of colloidal silver nanopaticles there was a greater synergistic effect on *S. aureus*. Although pyocyanine showed a slight antimicrobial effect on *C. albicans* and no antibacterial effect on *P. aeruginosa*, however in association with colloidal silver nanoparticles, pyocyanine had a synergistic effect on *C. albicans* and no effect on *P. aeruginosa* (Fig 6).

## Discussion

Despite an improvement in the survival of burn patients, infectious complications continue to be the major cause of morbidity and mortality (21). *P. aeruginosa* is one of the most important factors causing infection and mortality in these patients. In this study, we have isolated *P. aeruginosa* at a frequency of about 67.75% from burn patients at Shaheed Motahhari Burn Hospital, Tehran, Iran. This is in agreement with prior reports from other areas in Iran. In 2007, Ekrami et al. (21) isolated *P. aeruginosa* at a frequency of 37.5%. In recent years, the frequency of *P. aeruginosa* infections has increased in Iranian hospitals (21). These results are in accordance with studies in other countries (22-24). Out of 108 environmental samples, we have isolated 32 strains of *P. aeruginosa*, which corresponds to results of a study by Green et al. (25). They have shown that moderately high temperature and humidity (27℃ and 80%-95% humidity) favored colonization and survival of *P. aeruginosa* in environmental samples (25). Refinery soil contained more *P. aeruginosa* than other soil samples, possibly due to the rich carbon source in this soil. These isolates produced a more enhanced blue-green color on the medium cultures in a shorter amount of time. In addition to *P. aeruginosa*, microorganisms such as *Acinetobacter lwoffii, Aeromonas hydrophila, Flavobacterium*, and *Pseudomonas putida* have been isolated from contaminated soils that contain petroleum hydrocarbons and chemicals such as pesticides, thus showing the power of this bacterial enzyme (26-28).

In this study, 3 (6.25%) strains of the 48 clinical isolates of *P. aeruginosa* did not produce a blue-green color on nutrient, MH and cetrimide agars, while all 32 (100%) strains isolated from environmental sources did. In this research pyocyanine biosynthetic genes, which included the phenaA two modifying genes *(phzM, phzS)* were detected by PCR from clinical and non-clinical strains of *P. aeruginosa*. The protein products of *phzM* and *phzS* genes were detected by SDS-PAGE. All 32 (100%) bacteria isolated from environmental samples contained gene bands related to these genes. However 2 (4.17%; C10 and C6) out of 3 clinical strains did not produce a blue-green color, nor did they have 313 bp and 644 bp bands related to the *phzM* and *phzS* genes, respectively. These strains produced yellow and red colors on MH agar, but did not have 36.4 kDa and 43.6 kDa related to PhzM and PhzS proteins, respectively. Another strain (C7) despite the existence of the *phzM* gene, did not have its protein product and produced a yellow color. The results of this study were similar to findings of Mavrodi et al. (6).

There are two copies of the phenazine biosynthetic operon in *P. aeruginosa* which is the only Pseudomonas species that has two copies of this operon. The two *phz* operons are 98.3% identical at the DNA level and completely conserved. *P. aeruginosa* with insertionally inactivated *phzM* or *phzS* developed pyocyanine-deficient phenotypes. These strains produced yellow and red colors, respectively. When mutations were repaired, pyocyanine was reproduced. Research has shown that the activity of the PhzM and PhzS enzymes is necessary to produce pyocyanine (6).

**Fig 7:**
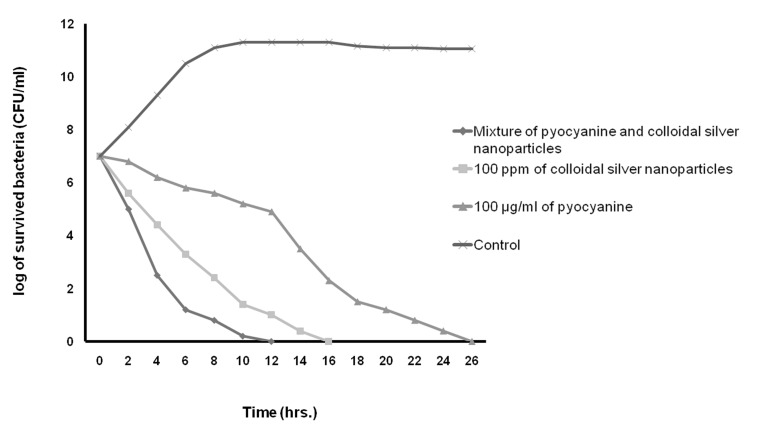
Kinetics of death rate of S. aureus in the presence of pyocyanine and colloidal silver nanoparticles compared to the mixture of pyocyanine and colloidal silver nanoparticles.

**Fig 8:**
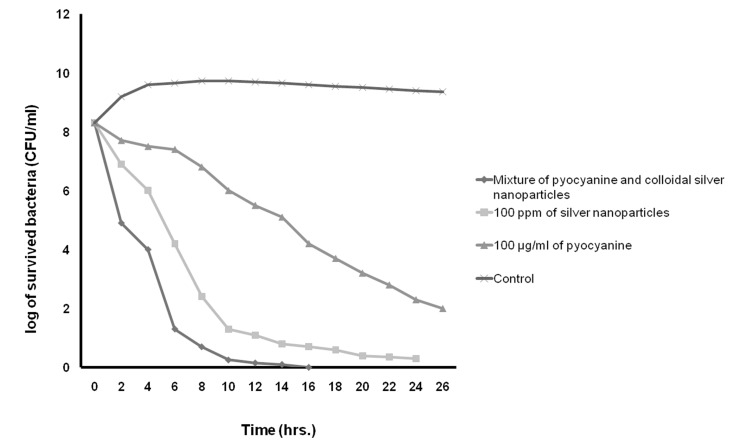
The kinetics of death rate of E. coli in the presence of pyocyanine and colloidal silver nanoparticles compared to the mixture of pyocyanine and colloidal silver nanoparticles.

**Fig 9:**
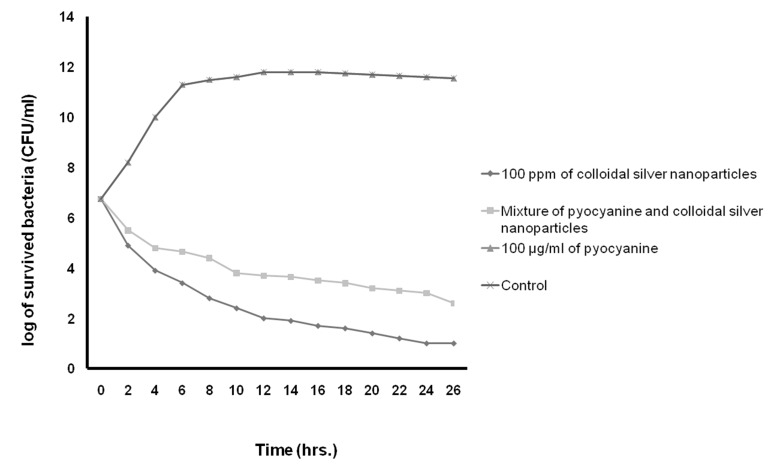
The kinetics of death rate of P. aeruginosa in the presence of pyocyanine and colloidal silver nanoparticles compared to the mixture of pyocyanine and colloidal silver nanoparticles

**Fig 10:**
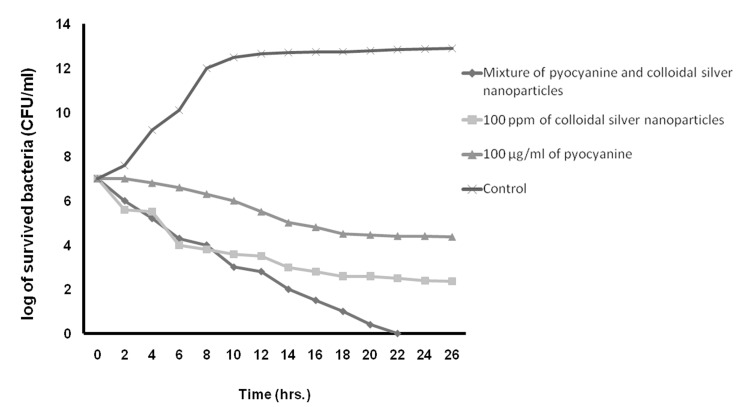
The kinetics of death rate of C. albicans in the presence of pyocyanine and colloidal silver nanoparticles compared to the mixture of pyocyanine and colloidal silver nanoparticles.

In this study, Table 3 shows the MICs, MBCs, and MFC of pyocyanine, colloidal silver nanoparticles and the pyocyanine-silver nanoparticle mixture against individually tested microbial strains and pyocyanine as an antimicrobial agent. The results agree with those by Ra’oof and Latif (29), Hasset et al. (30), and Baron et al. (15), which have shown the antimicrobial activity of pyocyanine. These results in conjunction with results of kinetics inhibition and disk, well and spot diffusion tests have shown that the pyocyanine antimicrobial effects are bactericidal in nature. Although in agreement with the study by Baron et al. (15), they contradict the results obtained by Waksman et al. (31). Baron et al. have established the concentration of dependent bactericidal action of pyocyanine, however Waksman et al. categorized pyocyanine as a bacteriostatic agent. Additionally, Baron et al. performed their experiments in liquid medium whereas Waksman et al. used agar plates. As seen in figure 9, the results in this study were similar to those obtained by Baron et al., where pyocyanine had no antimicrobial effects on *P. aeruginosa*, even at high concentrations of this pigment. Resistance of *P. aeruginosa* against pyocyanine is probably a genetic feature (15). Hassett et al. (30) have indicated that resistance of *P. aeruginosa* to pyocyanine is because of limited redox cycling of this compound and that under conditions favoring pyocyanine production, catalase and superoxide dismutase activities increase. Figure 9 also shows that silver nanoparticles mixed with pyocyanine did not have additional antimicrobial effects on *P. aeruginosa*. The MIC of pyocyanine on S. aureus and *E.coli* were similar, although kinetics of pyocyanine inhibition (Figs 7-10)

showed that the pigment was more effective against *S. aureus* growth than *E. coli*. The results of this study showed that the MIC of pyocyanine on *C. albicans* was about 100 µgml^-1^, the same as results by Kaleli et al. (32) and Kerr et al. (33). Pyocyanine, even at high concentrations, partially inhibited the growth of C. albicans. Pyocyanine decreased intracellular cAMP and AMP, therefore, the growth of C. albicans was reduced in patients' lungs with cystic fibrosis (33).

 According to table 3 and figures 7 to 10, the results of antimicrobial effects of colloidal silver nanoparticles on gram-positive *S. aureus* were more than observed with the gram-negative bacteria. These results agreed with the results of Nando and Sarvanan (34) and Cho et al. (9). Nando and Sarvanan demonstrated that silver nanoparticle synt are observed for this compound against *S. aureus*. had the most antimicrobial activity against methi In the present study, the increased antimicrobial effects of colloidal silver nanoparticles with pyocyanine were investigated. MIC, MBC, and MFC diameter and kinetic of the inhibition zone of pyocyanine with cillin-resistant *Staphylococcus aureus* (MRSA). Other researchers such as Puzio and Maliszewska (35), Sondi and Sondi-Salopek (8) and Kim et al. (36) found that *E. coli* was the most sensitive to silver nanoparticles. Kim et al. reported different antimicrobial properties of silver nanoparticles in bacteria due to differences in the membrane structures, particularly in the thick peptidoglycan layer in gram-positive bacteria (36). Nevertheless, the results of this study disaffirm it.

In the present study, the increased antimicrobial effects of colloidal silver nanoparticles with pyocyanine were investigated. MIC, MBC, and MFC diameter and kinetic of the inhibition zone of pyocyanine with and without silver nanoparticles against tested strains are shown in Tables 3 and 4, and Figures 7-10. The highest fold increases are observed for this compound against *S. aureus*. This is the first report that concerned enhancement of the activity of pyocyanine mixed with another antimicrobial agent, such as colloidal silver nanoparticles. However, some researchers such as Shahverdi et al. (37) have shown enhanced antimicrobial activities of silver nanoparticles in combination with other antibiotic agents (β-lactam, erythromycin, clindamycin). The synergistic interactions between silver nanoparticles and membrane-permeabilizing antimicrobial peptides has been investigated by Ruden et al. (38), who reported that permeabilization of the outer bacterial membrane by polymyxin B enhanced the intrinsic antibiotic effect of silver nanoparticles (38). Like Ruden et al., we also believe that permeability of the membrane of microorganisms by pyocyanine may enhance the intrinsic antibiotic effect of the silver nanoparticles.

The results of this study and similar findings by Mavrodi et al. (6) indicate that the presence of *phzM* and *phzS* genes is necessary to produce pyocyanine. Regarding the relationship between the production rate of pyocyanine and increased pathogenicity of *P.aeruginosa* (3,5), hopefully gathering information about declining factors related to pyocyanine production, disabling this gene or its protein product will assist in curing patients infected with these bacteria.

The increased antimicrobial effects of pyocyanine when mixed with colloidal silver nanoparticles, may enable its use as a hygienic, disinfectant material for hospitals and placesat risk for large numbers of microorganisms.However, an increase in the number of study samples would lead to more accurate results.

## Conclusion

Our results showed that some of the *P. aeruginosa* strains are unable to produce pyocyanine due to the lack of the *phzM* and *phzS* genes. Combination of pyocyanine and colloidal silver nanoparticles demonstrated a strong antibacterial activity against the bacterial strains.
